# MetQy—an R package to query metabolic functions of genes and genomes

**DOI:** 10.1093/bioinformatics/bty447

**Published:** 2018-06-05

**Authors:** Andrea S Martinez-Vernon, Frederick Farrell, Orkun S Soyer

**Affiliations:** 1 Synthetic Biology Centre for Doctoral Training; 2 School of Life Sciences; 3 Warwick Integrative Synthetic Biology (WISB) Centre, Life Sciences Building, University of Warwick, Coventry CV4 7AL, UK

## Abstract

**Summary:**

With the rapid accumulation of sequencing data from genomic and metagenomic studies, there is an acute need for better tools that facilitate their analyses against biological functions. To this end, we developed MetQy, an open–source **R** package designed for query–based analysis of functional units in [meta]genomes and/or sets of genes using the The Kyoto Encyclopedia of Genes and Genomes (KEGG). Furthermore, MetQy contains visualization and analysis tools and facilitates KEGG’s flat file manipulation. Thus, MetQy enables better understanding of metabolic capabilities of known genomes or user–specified [meta]genomes by using the available information and can help guide studies in microbial ecology, metabolic engineering and synthetic biology.

**Availability and implementation:**

The MetQy **R** package is freely available and can be downloaded from our group’s website (http://osslab.lifesci.warwick.ac.uk) or GitHub (https://github.com/OSS-Lab/MetQy).

## 1 Introduction

The advent of molecular biology has made the characterization and analysis of genomic sequences a key part of all areas of life sciences research. In the case of single–cell organisms, identification of specific functions within the genome directly influences our ability to assess their fitness in a given environment and their potential roles in biotechnology. Particularly, we should theoretically be able to translate genomic data into physiological predictions. Genomic databases are a pre-requisite for making such predictions, but their full use also requires computational tools that allow easy access and systematic analyses of the data.

The Kyoto Encyclopedia of Genes and Genomes (KEGG) is one of the oldest and most comprehensive collections of databases. Its primary aim has been the digitising of current knowledge on genes and molecules and their interactions (Kanehisa, 1997; Kanehisa and Goto, 2000) and it includes 16 databases and 3 sequence data collections (Kanehisa *et al.*, 2017). While these data can be analysed via different tools on the KEGG website, the existing web interface allows only specific retrieval of information and analyses. Furthermore, although the whole of the data can be downloaded via (paid) FTP access, the systematic analysis of these data in a user–defined manner remains difficult and developing computational analysis tools for this purpose remains a niche expertise that is still not available in many research labs.

There are several specific tools that make use of certain aspects of the KEGG data more available to a wider user-base. Examples include PICRUSt (Langille *et al.*, 2013), BlastKOALA and GhostKOALA (Kanehisa *et al.*, 2016), all of which focus on metagenomics data analysis. However, to our knowledge there are no tools that facilitate the analyses and information retrieval from KEGG with regards to studying the relationship between genomic data and physiological function. Therefore, we have developed MetQy, an open–source, easy–to–use and readily expandable **R** package for such analyses. MetQy uses the **R**–platform because it is commonly used among biologists, it is featured in undergraduate education, and it contains extensive statistical packages which are useful in subsequent data analyses.

MetQy was developed to readily interface between the KEGG orthology, module and genome databases and perform automated cross–analyses on them. It consists of a set of functions that allow querying genes, enzymes and functional modules across genomes and vice versa, thereby enabling better understanding of genotype–phenotype mapping in single–celled organisms and providing guidance for cellular engineering in synthetic biology. MetQy can be used ‘as-is’, since the relevant components of the KEGG databases (downloaded on 20/02/2018) are included within the package. The included KEGG data constitutes only part of the entire encyclopedia and is ‘hidden’ in the package so that direct access to the data is not possible, complying with KEGG licence. Users with a paid KEGG subscription can use MetQy parsing functions to update the data that the package uses. The MetQy package and GitHub wiki contain extensive documentation and usage examples for each function.

## 2 Software features

MetQy contains three main groups of software functions: data query, parsing and analysis and visualization. These are briefly described below. For more detailed information and usage examples, please see the package documentation and GitHub wiki.

### 2.1 Metabolic query functions

The *query* family of functions allows the user to query the KEGG data structures in a systematic (and automated) way. Users without FTP access can analyse the KEGG genome, module and ortholog databases indirectly by using this family of functions on built–in formatted KEGG data which is not directly accessible by the user. Additionally, these functions feature optional arguments that allow users to provide up–to–date data (by using the *parsing* functions on KEGG FTP data) or their own data structures, such as custom–made KEGG–style modules. Additional query functions can be readily developed by the users, allowing expansion of MetQy. MetQy features five query functions for key functional analyses.

*query_genomes_to_modules* calculates the module completeness fraction (*mcf*) given a set of genes or genomes. It returns a matrix showing the *mcf* for each module. The *mcf* calculation is based on block–based, logical KEGG module definition (see GitHub wiki). The function input is the modules to be queried (default is all KEGG modules) and the set of genes to be considered. The gene set can be provided either as a set of KEGG ortholog or Enzyme Commission (EC) numbers, or as genome identifier(s), with the latter case resulting in automatic retrieval of all genes for the genome(s).

While the implementation of *query_genomes_to_modules* function is similar to KEGG mapper [a web interface tool that performs a similar task (http://www.genome.jp/kegg/mapper.html; Kanehisa *et al.*, 2017)], there are several key features that are different. The KEGG Mapper’s web interface does not allow for module–specific evaluation nor for automation of the analysis. Our implementation allows for specific KEGG modules to be evaluated, given their ID, name and/or class. It also provides the capacity to determine the *mcf* of a module, rather than only identifying modules that are complete or that have one block missing. Finally, as EC numbers are widely used in systems biology, we used the KEGG orthology to translate the K number–based module definitions to EC number–based module definitions. This allows for module evaluation based on both K and EC numbers.

*query_module_to_genomes* determines the KEGG genome(s) that have user–specified module(s) that are complete above a *mcf* threshold (defaults to 1, i.e. complete). *query_gene_to_modules* determines those KEGG modules that feature specific user–specified gene(s). *query_genes_to_genomes* determines which KEGG genomes contain user-specified gene(s). *query_missingGenes_from_module* determines the missing gene(s) (K or EC numbers) that would be required to have a complete KEGG module within a genome (or gene set).

### 2.2 Parsing KEGG databases

MetQy comes with built–in data components of KEGG. It is, however, possible for users with FTP KEGG access to update these data components to their latest version. The MetQy *parsing* functions allow the production of the updated data, by formatting the relevant KEGG data files into **R** structures. They can also be used as stand–alone functions to introduce KEGG data into the **R** environment. All *query* functions have been designed to take in these updated data.

MetQy features two generic parsing functions that deal with the two main KEGG file types: files without extension (*parseKEGG_file*) and ‘.list’ files (*parseKEGG_file.list*). *parseKEGG_file.list* formats KEGG files containing a mapping between two KEGG database entries into binary matrices. For example, the mapping between K numbers and EC numbers is contained in the ‘ko_enzyme.list’ file and shows which K numbers correspond to which EC numbers. *parseKEGG_file* formats a KEGG database file into an **R** data frame by automatically detecting fields of the KEGG data and transforms these into variables. MetQy also contains file–specific functions that use these generic functions.

### 2.3 Analysis and visualization

The analysis and visualization family of functions are designed to facilitate the analysis primarily of the output of the *query_genomes_to_modules* function, which generates a matrix of *mcf* values for the genomes and modules analysed. There are three *analysis* and five *plot* (visualization) functions.

*analysis_pca_mean_distance_calculation* is designed to process the output of a principal component analysis (PCA) performed on the *mcf* matrix (this can be done for example by applying the **R** function *stats:: prcomp* function). It uses the resulting numeric matrix containing the principal components to calculate the mean Euclidean distance as a measure of spread or variation (of the data). This assumes that every row represents a multi-dimensional point (a genome in this case), with coordinates given in the corresponding columns. The mean Euclidean distance of *p* points is calculated by adding the computed pairwise Euclidean distance in *n* dimensions between all the points divided by the total number of distances.

*analysis_pca_mean_distance_grouping* takes in the numeric matrix resulting from performing a PCA on the *mcf* matrix and a factor, such as genus, to group the rows (genomes) of the matrix and uses the previous function (*analysis_pca_mean_distance_calculation*) to calculate the mean Euclidean distance for each group.

*analysis_genomes_module_output* takes in the *mcf* matrix (genomes and modules as rows and columns, respectively) and produces a series of analyses and generates a report automatically by default. These analyses comprise of: (i) reporting the number of genomes (data sets) and modules analysed, producing a (ii) heatmap of the *mcf* of all genomes and modules analysed, (iii) a boxplot of the *mcf* across all genomes for each module, (iv) a scatter plot of the SD of the *mcf* across all genomes for each module and (v) identifying any modules that have a constant (zero-variance) *mcf* across all genomes and producing a table. In addition, the function performs, for every factor group specified, the following analyses: (vi) group the genomes according to that factor and create a heatmap of the mean *mcf* for each module across the genomes that make up each group, (vii) carry out a PCA analysis on all the *mcf* data, showing the cumulative variance and generating a PC plot, (viii) visualize the PC plot with an overlay of the factor grouping and, finally, (ix) measure the within-group (per factor) variance, using the mean Euclidean distance as a proxy for spread.

*plot_heatmap* can be used to visualize the *mcf* calculated by the *query_genomes_to_modules* function as a colour mapped matrix (with genomes against modules). *plot_scatter_byFactors* allows the automatic grouping of data as determined by a factor and produces a scatter plot with groups overlaid by colour. *plot_scatter* is useful to visualize numerical data associated to data groups generated by a factor. This category–based visualization can be used to plot the SD for each module’s *mcf* or the mean Euclidean distance (see the analysis description above for more details). *plot_variance_boxplot* takes the *mcf* matrix and produces a boxplot for each module. *plot_sunburst* makes a hierarchical arrangement of categorical data, such as KEGG module classes, and represents it in a dart–style, where the inner ring contains the most general (highest level) information which can be divided into sub-categories (rings going outwards). The final ring represents the most specific level of information and can be coloured by either the counts of the data or an additional set of values provided by the user (refer to the GitHub wiki for more information).

## 3 Uses and applications

MetQy facilitates the general usability of the KEGG database and allows users to gain qualitative information about the functional capacity of a given organism or gene set. Anticipated uses of the tool include synthetic biology, where it can facilitate the design and guiding of metabolic engineering studies by identifying missing genes needed for an organism to have a complete KEGG module, and identifying KEGG genomes with desired metabolic capabilities. For systems biology applications, it allows identification of key physiological features of organisms and development of stoichiometric metabolic models by analysing module completeness in specific genomes and identifying transporter modules and carbon utilization routes in genomes. Finally, in microbial ecology, MetQy can allow species–function mappings in metagenomes and insights into functional capabilities of ecological groups by analysing the metabolic capacity of novel genomes from metagenomic studies. Organisms can be put into different functional groups, and the functional profiles of different environments compared.

### 3.1 Example of usage

**Fig. 1.**
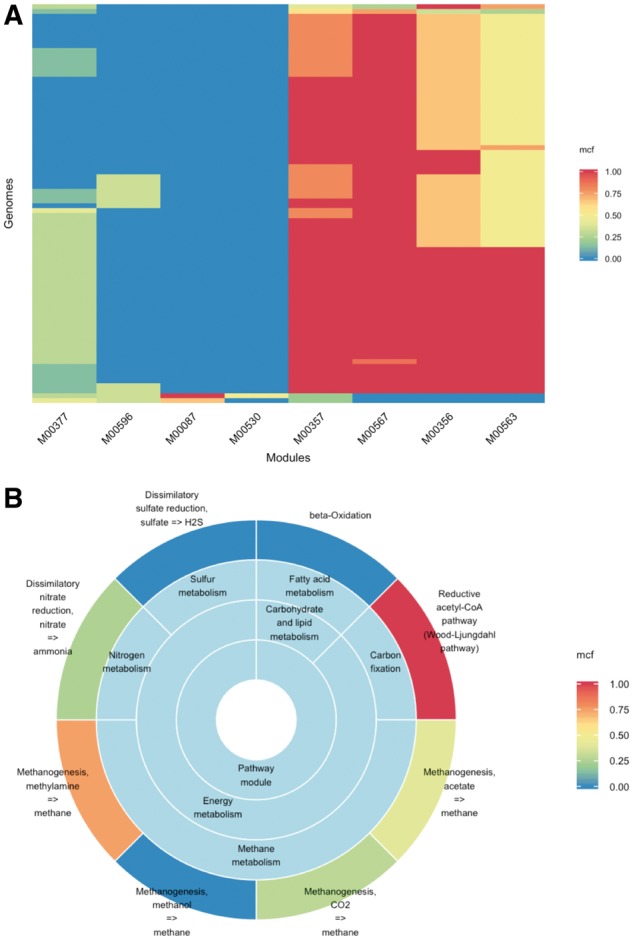
Visualization of some of the results obtained from an example analysis (Section 3.1). **(A)** Heatmap representation of module fraction completeness (*mcf*) across selected genomes (*y*–axis) and modules (*x*–axis). The *mcf* value is colour–coded as per the provided mapping scheme shown. **(B)** A sunburst diagram showing the mcf of different modules and their functional classes as obtained from the analysis of a specific genome (genome ID: T04272). The *mcf* value is colour–coded as per the provided mapping scheme shown. The data for both plots was obtained using MetQy function ‘query_genomes_to_modules’

To demonstrate some possible uses of MetQy functions, we have included a coded example on the MetQy GitHub wiki pages. This example demonstrates how MetQy can be used to retrieve KEGG genome data and how the metabolic functions of the extracted/matched organisms can be queried/identified in terms of KEGG modules. In the presented example, we evaluate the module completeness fraction (*mcf*) in methanogen genomes, focusing on sample KEGG modules loosely relating to the anaerobic digestion process (note that any user specified modules, or all KEGG can be used in a real analysis). We then visualize the results of this analysis as a heatmap using MetQy function *plot_heatmap* (Fig. 1A). In this example case, this analysis highlighted a specific module that is expected to be essential for methanogenesis (M00567: Methanogenesis, ${\text{CO}}_{2}=>$ methane) and that was almost fully complete in most genomes as expected (mcf>=0.75 in 96% of genomes), but incomplete in some genomes. This prompted us to analyse the genomes that had a lower *mcf* for this key module We thus identified the genome T04272 (Methanogenic archaeon ISO4-H5) as an interesting methanogen to focus on and used another MetQy function *plot_sunburst* to analyse all of its modules’ *mcf* through a sunburst plot (Fig. 1B). Furthermore, we identified the genes that were missing for that module to be complete (for that organism).

While this example highlights how specific MetQy functions can be utilized on their own to develop a specific analysis pipeline, it is also possible to use MetQy functions to perform an automated analysis on a set of genomes grouped by genus (or another grouping factor provided by the user, e.g. species or sample origin) and generate a comprehensive report in an automated fashion (see description for *analysis_genomes_module_output* function, the PDF report file in the GitHub repository and the worked–out example in the GitHub wiki).
